# A strategy for qualitative and quantitative profiling of glycyrrhiza extract and discovery of potential markers by fingerprint-activity relationship modeling

**DOI:** 10.1038/s41598-019-38601-y

**Published:** 2019-02-04

**Authors:** Yujing Zhang, Chao Wang, Fangliang Yang, Guoxiang Sun

**Affiliations:** 1 School of Pharmacy, Shenyang Pharmaceutical University, Shenyang, P. R. China; 2 School of Pharmaceutical Engineering, Shenyang Pharmaceutical University, Shenyang, P. R. China

## Abstract

This study was to evaluate the quality consistency of glycyrrhiza extract and to explore the possible anti-oxidant components in combination with chromatographic fingerprint and bioactivity evaluation. Characteristic fingerprints of glycyrrhiza extract samples from different sources were generated by high performance liquid chromatography with diode array detector (HPLC-DAD) and evaluated using hierarchical clustering and similarity analysis. Compared with the conventional qualitative similarity evaluation method, the averagely linear quantified fingerprint method had an important quantitative similarity parameter supported by quantitative analysis, which was recommended in the fingerprint evaluation. Antioxidant activities of the glycyrrhiza extract samples were determined by DPPH (2, 2-diphenyl-1-picryldrazyl) radical scavenging assays. In addition, the fingerprint-efficacy relationship was investigated by the chemical fingerprints and the anti-oxidant activities utilizing partial least squares model, which was capable of exploring and discovering the bioactive components of glycyrrhiza extracts. Therefore, the present study provided a powerful strategy to evaluate the holistic quality consistency of medicinal plant.

## Introduction

Licorice, called “Gan-Cao” in Chinese, is the dried roots and rhizomes of Glycyrrhiza species (Leguminosae family)^1^. Licorice has been officially listed in the Chinese pharmacopoeia (2015 edition), Japanese Pharmacopoeia (JP17) and United States Pharmacopeia (USP40-NF35) as a well-known and important medicinal plant, which was found in traditional Chinese medicine (TCM) prescriptions at the rate of 60%^2^. The Glycyrrhiza genus contains about 30 species, which are widely distributed all over the world^3^. However, only three species of Glycyrrhiza uralensis, G. glabra and G. inflate are officially used as Gan-Cao in Chinese Pharmacopoeia (2015 edition)^4^. Standardized licorice extract has been widely used in cosmetics, tobacco industry and sweets, as well as an additive to different beverages (brandy, liqueur, etc.) because of its sweet taste^5^. With pronounced anti-oxidant, anti-inflammatory, antiulcerogenic, antiviral, antitussive, immunostimulant, antispasmodic and antitumor properties^6^, licorice is used to treat allergic-inflammatory disease, cardiovascular disease, gastrointestinal problems, kidney ailments, as well as to prevent cancer^7,8^. These properties are attributed to the chemical components of licorice. So far, over 400 compounds have been isolated from Glycyrrhiza species^9^. Modern pharmacological studies showed extensively that flavonoids such as liquiritin apioside (LQA), liquiritigenin (LQG), isoliquiritoside (ISS), isoliquiritigenin (ISG) and liquiritin (LQT), triterpene saponins such as glycyrrhizic acid (GLA) were the main bioactive components underpinning the pharmacological effects of licorice^1,9,10^. Which were typically evaluated during quality control of licorice and its related products. GLA, a major triterpenoid saponin in licorice, is responsible for the antiviral activity and chemopreventive activity^11,12^. Conversely, LQT, a major flavonoid in licorice, has functional bioactivities including antidepressant-like effects, neuroprotective effects, and growth suppression of pathogenic intestinal bacteria^13–15^. Furthermore, the flavonoids can effectively inhibit macrophage mediated oxidation of low-density lipoprotein and attenuate lipopolysaccharide induced pulmonary inflammation^16^. In Chinese Pharmacopoeia (2015 edition), GLA and LQT were selected as the markers for the quality control analysis of licorice^4^. In fact, most published reports only quantify one or a limited number of components in licorice to control the quality. However, fluctuations in chemical composition and content depend on many factors, such as herbal species, anatomical part of the herbal medicines (HM), cultivating region, harvesting time and storage conditions, etc^17^. Therefore, these variables make it difficult for herbs to achieve quality consistency. Furthermore, HM herb is composed of many chemical components, and its therapeutic effects are confined to a separate and simple effect of a single bioactive component. Thence, effective and systematic quality control methods are necessary. The World Health Organization (WHO), US Food and Drug Administration (FDA), China Food and Drug Administration (CFDA) and European Medicine Agency (EMA) have all accepted the fingerprinting techniques and promote its use for the identification and quality control of complicated matrices^18^. Obviously, the fingerprinting technique, especially chromatography fingerprinting, has become a powerful tool for the quality control of HM. Several chromatographic techniques including thin-layer chromatography (TLC)^19^, fourier transform infrared spectroscopy (FT-IR)^20^, capillary electrophoresis (CE)^21^, gas chromatography (GC)^22^, liquid chromatography (LC)^2^ and high-speed countercurrent chromatography (HSCCC)^23^, have been applied to establish fingerprints of HM. Among these detection methods, HPLC is preferred due to its high reproducibility, sensitivity, adaptability for a wide range of samples.

Conventional chromatography fingerprinting methods are typically used in authenticity and identification^24,25^, which are mostly analyzed for qualitative similarity among the samples, and lack the quantitative evaluation of the fingerprints. The capability of fingerprinting in quality control of HM has been verified^26,27^, nevertheless, multi-component quantification is not credible in situations where certain medicinal ingredients are ignored. But this is not the case currently. Now, this stalemate has been broken. Quantitative similarity analysis (Averagely linear quantified fingerprint method, ALQFM) has recently been developed as a measure to detect the difference in the quantitative contents of powdered poppy capsule extractive by Zhang *et al*.^28^ and compound liquorice tablets by Zhang *et al*.^29^. In this study, ALQFM was used to the identify complex multi-component system (e.g., glycyrrhiza extract, GE) by qualitative similarity evaluation and secondly quantified GE samples from an overall mode. Simultaneously, the average linear quantitative similarity (${P}_{L}$) and the content of six bioactive components showed a high correlation, suggesting that ${P}_{L}$ was a low consumed and highly effective alternative to any multi-marker quantitative analysis. In addition, hierarchical clustering analysis (HCA) was also used for chromatographic fingerprint analysis to support the qualitative similarity assessment of GE samples with its strong discriminating ability. Nevertheless, chemical fingerprinting alone only improves quality control, and the key components of GE samples curing diseases are still unclear.

As is well known, the imbalance between the reactive oxygen species and production of antioxidant enzymes will induce oxidative stress^30,31^. Consequently, we should preferentially find natural, safe and effective antioxidants (food/medicine) that can protect the human body and slow the progress of many diseases, including cancer, which is associated with an increase in free radicals due to cell oxidation^32,33^. In addition, the development of rapid, quantitative and qualitative detection techniques is crucial. However, the batch DPPH assay has limitations such as high consumption, labour intensive and time consuming. Flow injection analysis (FIA) was ideally suited for automated analysis and had been successfully applied to rapid screening of food and pesticide^34–36^. As a kind of homologous medicine and food, licorice has high research value. The compositions of GE, including flavonoids, saponins and other compounds, have a common pharmacological activity, i.e., antioxidant activity^37–39^. In order to evaluate the antioxidant activity of licorice flavonoids, Liu *et al*.^40^ detected a significant decrease in the levels of three oxidative stress markers (NO, GSH and SOD) *in colon*, indicating that licorice flavonoids had strong antioxidant activities. So far, there has been no reports on the relationship between the chemical fingerprints of GE samples and their antioxidant activities, from any localities. Therefore, this could be a valuable study that contributes to the use in human nutrition and also helps prevent and treat diseases.

The present study was undertaken to characterize and quantify GE samples, as well as to assess their antioxidant potential. In addition, this study combined the chemical fingerprints and antioxidant activities utilizing partial least squares regression (PLS) model for exploring potential bioactive components in GE samples. The approach offered an effective and powerful way to evaluate the quality of GE samples.

## Materials and Methods

### Chemicals and materials

**Figure 1:**
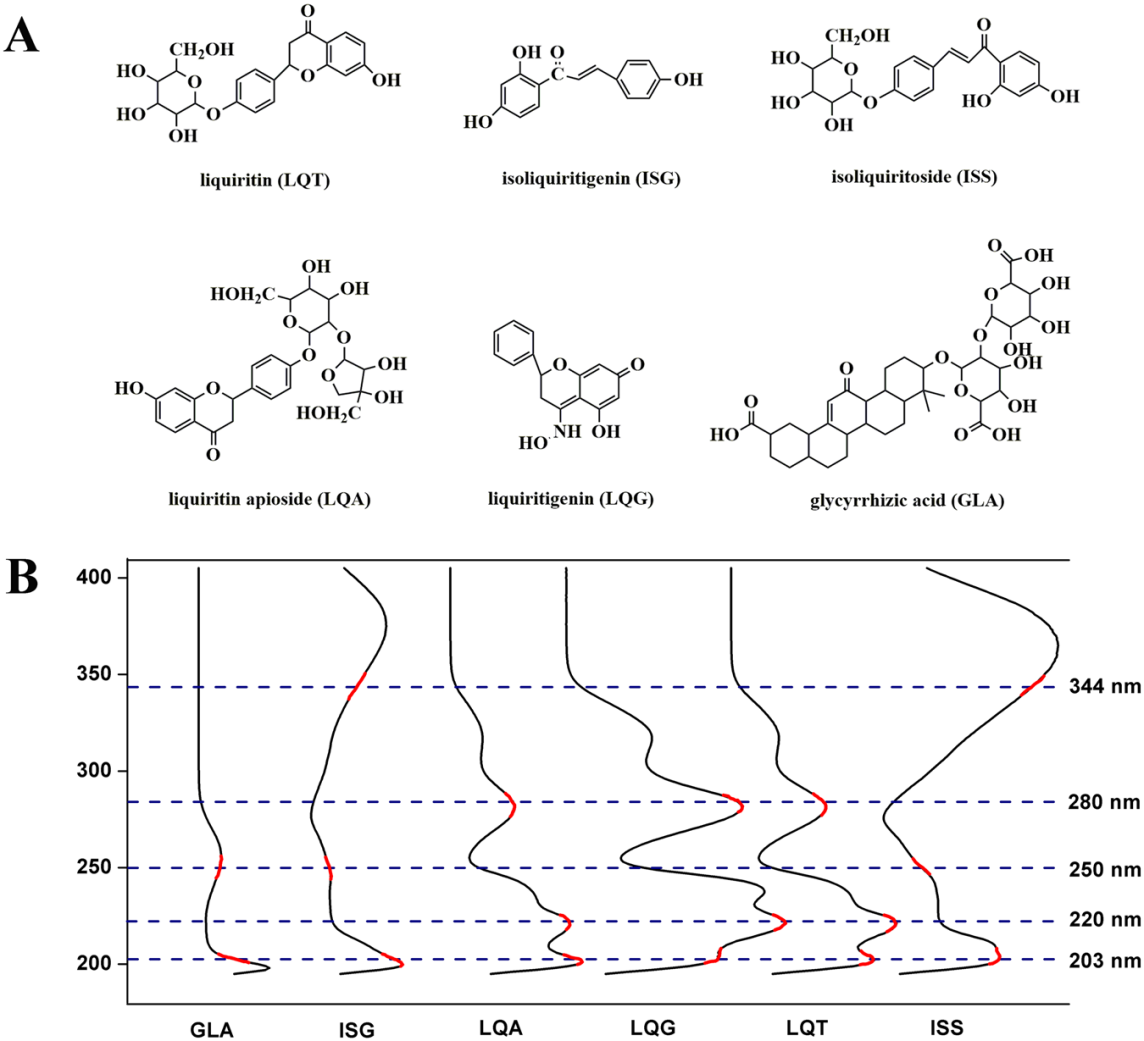
Chemical structures (**A**) and UV spectra (**B**) of six reference standards.

Phosphoric acid (HPLC-grade) was obtained from Kermel Chemistry Reagent Co., Ltd., Tianjin, China. Sodium 1-heptanesulfonate was supplied from Zhongmei Chromatographic Co., Ltd., Shandong, China. Acetonitrile, anhydrous ethyl alcohol and methanol (HPLC grade) were acquired from Shandong Yuwang Pharmaceutical Co., Ltd., Shandong, China. 1,1-diphenyl-2-picrylhydrazyl (DPPH) was provided from Sigma-Aldrich (Steinheim, Germany). All other chemicals and de-ionized water were of analytical grade. GE samples (S1-S30) were obtained from different manufacturers in the market (China), which were produced from Shanxi, China (Manufacturer A, including S1~S10) and Xinjiang, China (Manufacturer B, including S11~S20; Manufacturer C, including S21~S30), respectively. Reference standards of GLA, LQA, LQG, ISS and ISG were acquired from Shanghai Winherb Medical Technology Co., Ltd., Shanghai, China. LQT were supplied by the National Institutes for Food and Drug Control. The structures of the six investigated compounds were shown in Fig. 1A.

### Sample preparation and HPLC analysis

To obtain GE solutions, approximately 0.56 g of the GE sample was accurately weighed and transferred into a 50 mL volumetric flask. After addition methanol/water/ phosphoric acid (160:40:1, v/v/v), the flask was transferred into an ultrasonic cleaning bath for extraction for 20 min at 45 °C. Afterward, the supernatant was filtered through a 0.45-µm Millipore membrane and the filtrate was collected as sample solution. The standards solution was prepared by accurately weighed amount of GLA, LQG, LQA, LQT, ISS and ISG and dissolved with methanol, respectively. All solutions were stored at 4 °C in the dark until analysis.

Chromatographic separation was measured with an Agilent series 1100 system equipped with quaternary gradient pump, thermostatic column compartment, DAD, autosampler and a CAPCELL PAK C18 MG column (250 × 4.6 mm, 5.0 μm, shiseido, Japan). The mobile phase consisted of aqueous solution containing 0.1% (v/v) phosphoric acid and 5 mM sodium 1-heptanesulfonate (A) and acetonitrile containing 10% (v/v) anhydrous ethyl alcohol and aqueous solution and 2.4% (v/v) phosphoric acid (B). Separation was achieved using the following linear gradient program: 6–18% B at 0–10 min, 18–33% B at 10–20 min, 33–46% B at 20–32 min, 46–60% B at 32–45 min, 60–78% B at 45–60 min, 78–80% B at 60–65 min. The column temperature was maintained at 35 °C. A 10 μL aliquot of each sample was injected into the HPLC-DAD system. The flow rate was set at 1.0 mL/min. The detection wavelengths were set at 344 nm, 280 nm, 250 nm, 220 nm and 203 nm.

### Similarity analysis

**Figure 2:**
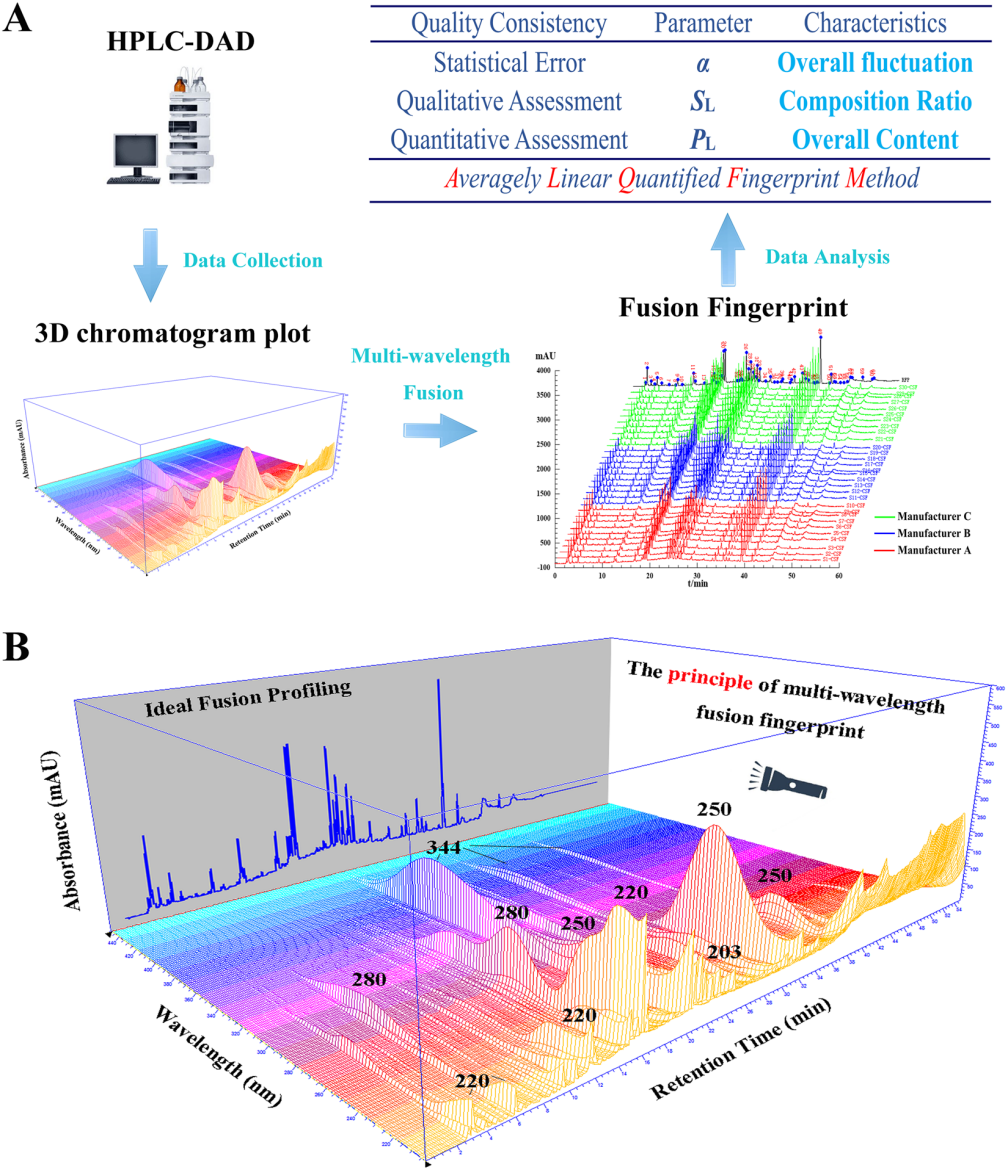
The visualization analysis process for ALQFM (**A**) and the principle of multi-wavelength fusion fingerprint (**B**).

ALQFM is a mathematical data processing method based on fingerprint vector, which contains the quality and quantity analysis of fingerprints. Meanwhile, the quality level of evaluation criteria can be applied to read the evaluation results more intuitively and easily. The visualization analysis process for ALQFM was shown in Fig. 2A.

$\overrightarrow{x}=({x}_{1},{x}_{2}\cdots {x}_{n})$ and $\overrightarrow{y}({y}_{1},{y}_{2}\cdots {y}_{n})$, where *x*_*i*_ and *y*_*i*_ are the *i*th peak area, serve as sample and reference fingerprint vector, respectively. Average linear qualitative similarity (${S}_{L}$, Eq. 1) can clearly reflect the degree of similarity in the chemical compositions of the sample fingerprint and reference fingerprint in terms of distribution ratio. To describe apparent and calculated similarity, slope of linear equation (***b***, Eq. 2) and apparent content similarity ($R \%$, Eq. 3) are calculated after weight-corrected by *m*_*S*_ (the weight of each GE sample) and *m*_*R*_ (the average weight of 30 GE samples). The closer $R \%$ and *b* are to 1, the more similar the sample fingerprint vector is to the reference fingerprint vector. Fingerprint variation coefficient ($\alpha$, Eq. 4), a statistical error, is calculated using $R \%$ and ***b***, which can reflect the accuracy of the model. Average linear quantitative similarity (${P}_{L}$, Eq. 5), an important quantitative parameter, is employed to monitor the overall content of all fingerprint components in the sample fingerprint. Accordingly, $\alpha$, ${S}_{L}$ and ${P}_{L}$ are combined in the averagely linear quantified fingerprint method (ALQFM) to evaluate the quality of TCM (8 grades listed in Supporting Information Table S1). Generally, samples with the grade ≤ 5 were recommended as the qualified ones.

1 $${S}_{L}=\frac{1}{2}(\frac{\sum _{i=1}^{n}({x}_{i}-\overline{{x}_{i}})({y}_{i}-\overline{{y}_{i}})}{\sqrt{\sum _{i=1}^{n}{({x}_{i}-\overline{{x}_{i}})}^{2}}\sqrt{\sum _{i=1}^{n}{({y}_{i}-\overline{{y}_{i}})}^{2}}}+\frac{\sum _{i=1}^{n}\frac{{x}_{i}}{{y}_{i}}}{\sqrt{n\sum _{i=1}^{n}{(\frac{{x}_{i}}{{y}_{i}})}^{2}}})$$

2 $$b=\frac{n\sum _{i=1}^{n}{x}_{i}{y}_{i}-\sum _{i=1}^{n}{x}_{i}\sum _{i=1}^{n}{y}_{i}}{n\sum _{i=1}^{n}{y}_{i}^{2}-\sum _{i=1}^{n}{y}_{i}^{2}}\times \frac{{m}_{R}}{{m}_{S}}\times 100 \% $$

3 $$R \% =\frac{\sum _{i=1}^{n}{x}_{i}}{\sum _{i=1}^{n}{y}_{i}}\times \frac{{m}_{R}}{{m}_{S}}\times 100 \% $$

4 $$\alpha =|\frac{R}{b}-1|$$

5 $${P}_{L}=\frac{1}{2}(\frac{\sum _{i=1}^{n}({x}_{i}-\overline{{x}_{i}})({y}_{i}-\overline{{y}_{i}})}{\sqrt{\sum _{i=1}^{n}{({x}_{i}-\overline{{x}_{i}})}^{2}}\sqrt{\sum _{i=1}^{n}{({y}_{i}-\overline{{y}_{i}})}^{2}}}\times b+\frac{\sum _{i=1}^{n}{x}_{i}\sum _{i=1}^{n}{x}_{i}{y}_{i}}{\sum _{i=1}^{n}{y}_{i}\sqrt{\sum _{i=1}^{n}{x}_{i}^{2}}\sqrt{\sum _{i=1}^{n}{y}_{i}^{2}}}\times 100 \% )$$

### Antioxidant activity assay

The radical scavenging property was measured on HPLC system (HPLC-1) mentioned in “*2*.*2 sample preparation and HPLC analysis*” section at a flow rate of 0.4 mL/min. 1 μL GE sample carried by mobile phase (A/B, 50/50, v/v) reached to a hollow polytetrafluoroethylene pipe (PTFE, 5000 mm × 0.18 mm i.d. from Agilent), where the 0.127 mM methanol DPPH solution was delivered via another HPLC system (HPLC-2, Agilent 1100 series) at a flow rate of 0.3 mL/min. Then the negative absorbance was measured with the UV-vis DAD at 517 nm. The test solution was performed in triplicate. Ascorbic acid, known as a well-known antioxidant, was used as a standard antioxidant. Thus, ascorbic acid equivalent (ASAE) was calculated to evaluate the antioxidant activity of GE samples. The linear range was 0.030–0.450 mM (*y* = 10168*x* + 10.273, *r* = 0.9992). The higher the ASAE value, the stronger the antioxidant activity.

## Results and Discussion

### Methodology validation of HPLC analysis

The calibration curve, including linear ranges as well as the limit of quantity (LOQ) and limit of detection (LOD), regression equation and correlation coefficient (R^2^) were showed in Supporting Information Table S2. All the analytes showed excellent linearity (R^2^ ≥ 0.9999) over the tested concentration ranges. The LOQ and LOD, which were determined in the range of 0.500–5.000 μg/mL and 0.025–0.125 μg/mL, respectively. The accuracy of the HPLC method was evaluated by using the standard addition method and the average percent recoveries for six investigated compounds ranged from 97.65% to 100.71%, with relative standard deviation (RSD) value less than 1.92%. RSD values of each co-possessing fingerprint peak area were, respectively, less than 3.40%, 1.65% and 2.78% for the stability, precision and repeatability tests. Considering these results, the method was accurate and valid enough.

### Compounds content analysis

The content of active ingredient contents in 30 GE samples was simultaneously determined using the established calibration curves (Supporting Information Table S2). Based on quantitative results (Table 1), GLA was found to be the main component in all the GE samples with the average values of 82.798 mg/ g. A large fluctuation in the content of LQG and LQA was observed in 30 GE samples as reflected by RSD of 36.299% for LQG and 36.091% for LQA, respectively. The high RSD value was due to the low levels of LQG and large variation in content of LQA between different manufacturers. Based on analysis of variance (ANOVA), performed by SPSS statistics software (SPSS 19.0 for Windows, SPSS Inc., USA), there was no significant difference in the content of the six components in each manufacturer (P > 0.05). However, LQA, LQG and ISS showed significant differences between Manufacturer 1 and Manufacturer 3; LQA and GLA showed significant differences between Manufacturer 1 and Manufacturer 2 (P < 0.05). The possible explanation for the variation of the contents could be the variability in herbal processes or the regionalization of raw herbs caused by many factors^17,41,42^.

**Table 1:** The results of the compounds content analysis and overview of the experimental and predicted values of PLS model.

Sample (mg/g)	LQA	LQT	LQG	GLA	ISS	ISG	*P*_6C_%	Var. ASAE (mM)	Pred. ASAE (mM)	RE^c^ (%)
S1^a^	8.470	10.034	1.621	78.446	4.040	2.263	84.204	0.2178	0.2148	−1.37
S2^a^	8.097	9.754	1.574	73.001	3.723	2.100	79.435	0.2110	0.2030	−3.78
S3^b^	8.714	9.659	1.726	77.484	3.712	2.433	84.002	0.2247	0.2078	−7.54
S4^a^	9.657	10.926	1.875	82.536	3.700	2.305	87.990	0.2335	0.2293	−1.82
S5^a^	9.624	10.892	1.884	80.195	3.584	2.308	86.982	0.2197	0.2203	0.31
S6^d^	8.085	13.722	3.670	60.747	4.427	3.790	111.528	0.1365		
S7^b^	9.296	12.003	2.053	85.557	4.054	2.418	93.451	0.2120	0.2170	2.36
S8^a^	9.817	11.781	1.904	83.763	4.112	2.170	90.903	0.2232	0.2228	−0.16
S9^a^	8.080	8.412	1.951	79.059	3.521	2.755	84.559	0.1594	0.1881	18.01
S10^a^	8.972	11.401	2.041	76.784	4.394	2.727	93.992	0.1984	0.1923	−3.07
S11^a^	21.731	10.675	2.114	93.908	3.251	2.609	103.750	0.2793	0.2789	−0.16
S12^a^	24.364	11.781	2.068	95.158	3.589	2.285	107.459	0.2809	0.2804	−0.17
S13^b^	22.511	10.878	2.319	93.076	3.346	2.689	107.034	0.2504	0.2859	14.21
S14^a^	24.314	12.040	1.847	88.789	3.457	1.846	101.505	0.2804	0.2809	0.17
S15^a^	20.247	11.478	2.276	95.474	3.485	2.196	103.197	0.2458	0.2591	5.44
S16^a^	20.908	10.021	2.361	88.114	3.561	3.171	107.585	0.2489	0.2530	1.64
S17^b^	18.255	9.232	2.019	85.626	3.545	2.464	96.126	0.2148	0.2577	20.00
S18^d^	13.703	11.014	5.181	77.198	4.409	4.573	131.753	0.2046		
S19^b^	14.741	11.101	2.485	82.361	4.174	3.134	105.205	0.1753	0.2238	27.62
S20^a^	20.158	8.646	1.506	79.109	3.216	1.828	86.665	0.2494	0.2329	−6.62
S21^a^	19.043	11.877	3.193	75.550	3.236	1.669	100.078	0.2250	0.2280	1.35
S22^a^	21.414	13.321	3.362	87.217	3.520	1.717	109.792	0.2380	0.2354	−1.13
S23^b^	21.846	13.897	3.412	89.752	3.596	1.858	113.235	0.2316	0.2482	7.19
S24^a^	22.441	13.670	2.817	85.938	3.788	2.004	110.587	0.2555	0.2604	1.90
S25^a^	15.160	10.320	4.952	87.349	2.959	3.292	117.473	0.2436	0.2372	−2.64
S26^a^	20.717	11.902	2.572	76.887	3.158	1.946	99.394	0.2318	0.2394	3.26
S27^b^	22.361	10.317	2.444	77.354	2.969	2.654	101.738	0.2291	0.2310	0.85
S28^a^	20.628	11.224	2.822	89.800	3.272	2.816	108.860	0.2488	0.2503	0.62
S29^a^	17.293	9.856	1.792	76.981	3.137	2.170	88.978	0.2352	0.2210	−6.02
S30^a^	20.202	9.227	2.517	80.700	3.108	3.084	102.551	0.2360	0.2341	−0.82
Mean	16.362	11.035	2.479	82.798	3.601	2.509	100.000	0.2321	0.2369	2.49
RSD%	36.091	13.007	36.299	9.131	11.666	25.504	11.877			

LQA, liquiritin apioside; LQT, liquiritin; LQG, liquiritigenin; GLA, glycyrrhizic acid; ISS, isoliquiritoside; ISG, isoliquiritigenin; *P*_6C_%, the average content of the six compounds.

^a^Used for the calibration model.

^b^Used for the prediction model.

^c^RE, relative error.

^d^Outliers.

### HPLC fingerprint analysis

**Figure 3:**
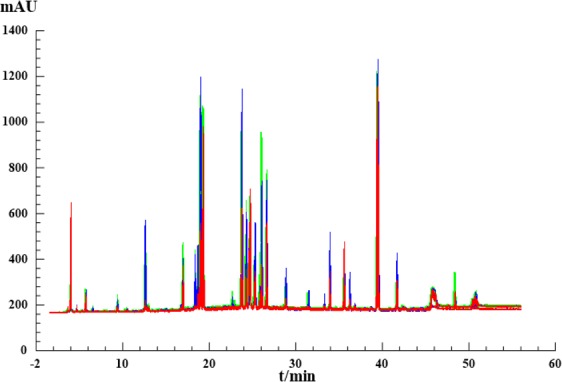
Overlaid diagram of Fusion fingerprints of 30 batches of GE samples.

The validated HPLC method was successfully used to generate fingerprints of the GE samples, presented in Supporting Information Fig. S1. Six investigated compounds show different UV absorption as illustrated in Fig. 1B. It is reasonable to believe that the ingredients in the GE samples also have very different absorption behavior. According to previous reports, UV spectra of six reference standards (Fig. 1B) and 3D chromatogram plot (Fig. 2B), five strong absorption bands (203, 220, 250, 280 and 344 nm) that contained 39, 39, 44, 30 and 19 co-possessing peaks (Supporting Information Fig. S1), respectively, corresponding to the flavonoids and triterpene saponins^29^. In order to perform quality control of HM, it is necessary to establish a chromatogram with comprehensive composition information. However, for the complexity of HM compositions, it is difficult to accomplish this at a single wavelength. Therefore, fusion fingerprint^43^ capable of synthesizing enhancing signal response and rich fingerprint information was absolutely necessary. As shown in Fig. 3, typical fusion HPLC fingerprints of 30 GE samples had similar chemical profiles with some common chemical compositions. Next, GE sample fingerprints and the reference fingerprint (RFP constructed by taking the average of the GE sample chromatograms) were imported to an in-house software “Digitized Evaluation System for Super-Information Characteristics of TCM Chromatographic Fingerprints 4.0” (Software certificated NO. 0407573, China) to calculate the quality evaluation results as presented in Table 2.

**Table 2:** The evaluation results obtained from ALQFM of 30 batches of GE samples from three different manufacturers.

Sample	203 nm				220 nm				250 nm				280 nm				344 nm				Fusion			
	*S*_L_	*P*_L_%	*α*	Grade	*S*_L_	*P*_L_%	*α*	Grade	*S*_L_	*P*_L_%	*α*	Grade	*S*_L_	*P*_L_%	*α*	Grade	*S*_L_	*P*_L_%	*α*	Grade	*S*_L_	*P*_L_%	*α*	Grade
S1	0.914	56.1	0.105	7	0.939	69.8	0.080	6	0.952	94.8	0.018	2	0.914	82.0	0.068	4	0.958	76.2	0.025	5	0.927	76.4	0.015	5
S2	0.889	54.2	0.093	7	0.944	67.2	0.063	6	0.969	88.1	0.004	3	0.925	78.1	0.052	5	0.957	69.8	0.023	6	0.945	73.6	0.014	5
S3	0.910	55.6	0.181	7	0.941	69.5	0.071	6	0.963	88.6	0.119	3	0.919	80.6	0.067	4	0.957	73.0	0.007	5	0.943	75.1	0.040	5
S4	0.900	86.5	0.178	4	0.946	78.5	0.070	5	0.971	97.1	0.068	2	0.922	83.2	0.048	4	0.961	71.5	0.021	5	0.944	88.3	0.021	3
S5	0.925	87.4	0.149	3	0.948	77.4	0.054	5	0.973	94.2	0.082	2	0.910	81.4	0.046	4	0.961	69.9	0.008	6	0.943	85.1	0.029	3
S6	0.816	73.7	0.088	5	0.884	71.5	0.036	5	0.960	74.6	0.051	5	0.870	73.6	0.029	5	0.897	72.0	0.059	5	0.899	76.1	0.010	5
S7	0.864	85.1	0.108	3	0.932	77.5	0.032	5	0.979	99.2	0.119	3	0.901	85.7	0.083	3	0.950	74.5	0.029	5	0.936	88.8	0.053	3
S8	0.892	88.5	0.093	3	0.941	81.1	0.041	4	0.963	101.8	0.036	1	0.927	84.2	0.010	4	0.959	75.7	0.035	5	0.942	91.9	0.010	2
S9	0.885	48.3	0.126	8	0.925	66.3	0.089	6	0.964	94.9	0.014	2	0.892	80.1	0.064	4	0.916	66.6	0.081	6	0.915	74.5	0.046	5
S10	0.894	54.4	0.085	7	0.926	71.3	0.016	5	0.983	91.0	0.057	2	0.917	89.5	0.111	3	0.950	78.7	0.000	5	0.944	77.1	0.044	5
S11	0.953	133.2	0.113	6	0.976	118.4	0.002	4	0.989	112.2	0.029	3	0.979	116.1	0.000	4	0.990	110.6	0.011	3	0.969	114.7	0.032	3
S12	0.965	139.1	0.089	6	0.976	122.8	0.066	5	0.978	116.4	0.051	4	0.975	125.0	0.059	5	0.981	123.0	0.063	5	0.960	124.2	0.020	5
S13	0.949	139.2	0.015	6	0.968	120.5	0.009	5	0.985	112.1	0.003	3	0.975	120.9	0.053	5	0.987	119.7	0.023	4	0.969	122.7	0.024	5
S14	0.946	131.2	0.123	6	0.967	120.6	0.076	5	0.987	106.2	0.021	2	0.969	120.2	0.088	5	0.974	118.6	0.089	4	0.963	113.9	0.054	3
S15	0.916	129.0	0.075	5	0.969	116.2	0.014	4	0.964	118.4	0.077	4	0.964	103.5	0.002	1	0.961	97.4	0.013	1	0.942	116.3	0.014	4
S16	0.931	78.5	0.040	5	0.957	106.3	0.032	2	0.976	105.7	0.006	2	0.969	120.2	0.078	5	0.954	131.4	0.098	6	0.954	103.1	0.034	1
S17	0.942	81.1	0.002	4	0.974	100.9	0.023	1	0.987	103.8	0.012	1	0.979	115.3	0.005	4	0.988	117.4	0.056	4	0.972	100.5	0.014	1
S18	0.836	66.2	0.273	6	0.865	95.1	0.311	6	0.942	98.7	0.230	5	0.848	100.2	0.335	6	0.876	105.8	0.233	5	0.903	95.9	0.197	4
S19	0.950	65.3	0.118	6	0.979	93.1	0.002	2	0.977	100.5	0.025	1	0.982	104.3	0.005	1	0.948	96.2	0.060	2	0.960	91.3	0.003	2
S20	0.940	71.9	0.038	5	0.968	96.5	0.071	2	0.985	95.3	0.006	1	0.952	103.0	0.128	3	0.957	117.9	0.059	4	0.961	93.3	0.043	2
S21	0.944	107.0	0.031	2	0.976	105.6	0.084	2	0.954	91.8	0.058	2	0.917	80.1	0.035	4	0.971	97.7	0.031	1	0.946	96.5	0.001	2
S22	0.947	127.5	0.043	5	0.978	116.0	0.094	4	0.919	105.2	0.084	2	0.919	92.2	0.000	2	0.976	106.1	0.037	2	0.930	110.9	0.000	3
S23	0.953	123.4	0.047	5	0.973	119.4	0.116	4	0.976	105.3	0.062	2	0.917	91.7	0.005	2	0.975	109.3	0.033	2	0.953	106.6	0.082	2
S24	0.952	135.4	0.064	6	0.968	121.4	0.046	5	0.969	102.0	0.031	1	0.926	96.4	0.009	2	0.950	118.9	0.035	4	0.956	114.3	0.015	3
S25	0.944	116.8	0.058	4	0.950	98.7	0.073	2	0.978	102.8	0.058	2	0.948	90.1	0.115	3	0.979	87.4	0.051	3	0.964	103.3	0.037	1
S26	0.951	116.3	0.104	4	0.973	109.1	0.076	2	0.964	93.3	0.042	2	0.921	85.5	0.054	3	0.976	100.6	0.012	1	0.954	101.0	0.003	1
S27	0.928	132.7	0.071	6	0.952	112.1	0.027	3	0.981	93.7	0.018	2	0.906	85.2	0.117	3	0.977	108.9	0.049	2	0.949	107.2	0.032	2
S28	0.928	125.1	0.059	5	0.967	116.0	0.013	4	0.983	106.8	0.040	2	0.915	87.3	0.218	5	0.982	105.7	0.053	2	0.960	113.2	0.001	3
S29	0.949	73.1	0.052	5	0.965	95.1	0.001	1	0.981	92.6	0.008	2	0.934	87.2	0.063	3	0.974	101.8	0.088	2	0.955	89.9	0.023	3
S30	0.935	78.7	0.037	5	0.961	103.5	0.013	1	0.982	98.1	0.030	1	0.894	91.8	0.101	3	0.961	115.8	0.072	4	0.952	97.7	0.016	1
RFP	1.000	100.0	0.000	1	1.000	100.0	0.000	1	1.000	100.0	0.000	1	1.000	100.0	0.000	1	1.000	100.0	0.000	1	1.000	100.0	0.000	1

Table 2 showed that except S6 (grade 5), the fusion fingerprint grades exhibited some fluctuations compared with the results at each wavelength. The resulting fluctuations may be caused by differences in response intensity and number of fingerprints at each wavelength. Consequently, the fusion fingerprint evaluation strategy is comprehensive and indispensable for avoiding potential deviations from single wavelength. Furthermore, except S18, the remaining 29 GE samples had $\alpha$ < 0.30 and all of the 30 GE samples had ${S}_{L}$ > 0.80, indicating that the samples were similar in the distribution and number of chemical compositions. Based on the qualitative parameters ${S}_{L}$ and $\alpha$, the quality grades of 29 samples should be within 1–5. In fact, at 203 nm, S9 and S13 were judged to be outliers (8 and 6 respectively) combined with quantitative parameters (48.3% and 139.2%, respectively). For fingerprint analysis, a qualitative assessment (an acceptable $\alpha$ < 0.30 and ${S}_{L}$ > 0.70) should be performed firstly, followed by a further quantitative evaluation (acceptable 70.0% ≤ ≤ 130.0%). Eventually, in this study, the fusion qualities of 30 GE samples were successfully evaluated in terms of the criteria (shown in Table 2). The quality grade of S16, S17, S25, S26, S30 were best (Grade 1), those of S8, S19-S21, S23 and S27 were better (Grade 2), and those of S4, S5, S7, S11, S14, S22, S24, S28 and S29 were good (Grade 3), and those of S15 and S18 were fine (Grade 4), except for that of S1-S3, S6, S9, S10, S12 and S13 as moderate (Grade 5) due to their much lower or higher contents. ALQFM provides a reliable and feasible strategy to qualitatively and quantitatively assess TCM/HM fingerprints simultaneously.

### Correlation between average linear quantitative similarity and quantitative analysis

In “*Compounds content analysis*” section, the compounds content in the GE samples were accurately quantitated using chemical reference substances. However, available chemical reference substances and more time were required for the quantitative analysis. Even if quantitation is feasible when the chemical reference substances are available, the ingredients of TCM/HM are too complex to accurately control their quality based on the quantitative analysis completely. Therefore, quantitative fingerprint becomes more critical if it is consistent with the compounds content analysis.

As discussed in “*HPLC fingerprint analysis*” section, ${P}_{L}$ is an important parameter for quantifying all fingerprint components in the sample fingerprint. The relationship between ${P}_{L}$ and the quantitative content of the six compounds was further explored. The ***P***_**6C**_ values (*y*, average of six component percentages, Table 1) were, respectively, plotted *vs*. the ${P}_{L}$ values (*x*, ***P***_**L**_−203 nm, ***P***_**L**_−220 nm, ***P***_**L**_−250 nm, ***P***_**L**_−280 nm, ***P***_**L**_−344 nm and ***P***_**L**_-fusion, Table 2) as shown in Supporting Information Fig. S2. Linear regression shown that the correlation coefficients (*r*) were all above 0.5435, especially the correlation coefficient between ***P***_**6C**_ and ***P***_**L**_-fusion, reached an excellent value of 0.8487. This indicated that ${P}_{L}$ was basically consistent with the quantitative results for the six investigated compounds. Consequently, due to the economic, reliability, feasibility and simplicity advantages of quantifiable fingerprints for HM quality control, which has the potential to replace multi-component quantification.

### Hierarchical clustering analysis

**Figure 4:**
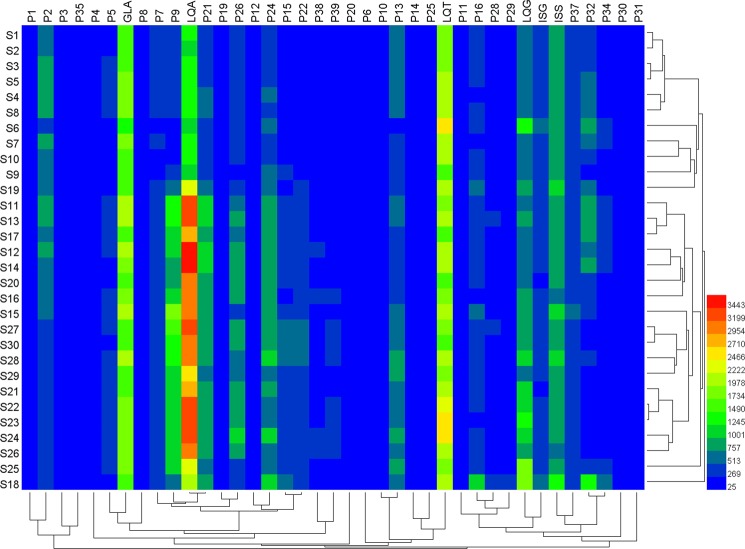
Hierarchical clustering analysis (HCA) of GE samples from three different manufacturers. S1~S10, manufacturer (**A**) S11~S20, manufacturer (**B**) S21~S30, manufacturer (**C**).

To investigate the internal characteristics of GE samples from different sources, HCA^44^, a powerful multivariate analysis technique, was performed on HPLC fingerprints. HCA was performed using HemI statistics sofware (Heatmap Illustrator, Version 1.0). For the 30 GE samples, the quality grade difference between the fusion and 220 nm results were all within two grades (Supporting Information Fig. S3). Therefore, 220 nm fingerprint was the closest one to the fusion fingerprint and reflected more compound information than any other wavelengths from both qualitative and quantitative aspects. In this study, HCA was performed according to the peak areas of the 39 co-possessing peaks of 30 GE samples at 220 nm. Subsequently, between-groups linkage method and squared Euclidean distance was used as average linkage and a metric to evaluate 30 GE samples similarity. A hierarchical cluster heat-map (Fig. 4) was obtained to represent the dissimilarity (between the different groups) or similarity (within the same group) between 30 GE samples. Two main clusters were produced (Samples S1-S10 collected from Manufacturer A, Shanxi province and S11-S20, S21-S30 collected from Manufacturer B, C, Xinjiang province). The distance between samples in the different cluster was farther than the distance between samples in the same cluster, suggesting that the internal quality characterized by HPLC chemical fingerprints varied widely between different clusters. From the above results, GE samples obtained from different geographic environments, such as different soil qualities, longitude, latitude and cultivating climates, had different internal qualities^17,41,42^. These results are consistent with the results of the quantitative fingerprint analysis, indicating that HCA only performs simple classification of samples, and the quantitative fingerprint analysis could provide a more accurate, feasible and reliable evaluation of TCM/HM. Subsequently, a simple and intuitive comparison of the chemical fingerprints (Fig. 3) involved comparing the color changes between the 39 co-possessing peaks in Fig. 4, which might be related to their different biological activities. In particular, the six investigated compounds showed strong color reactions. Thence, in the next part, antioxidant activity of the GE sample was studied.

### Antioxidant activity

#### Antioxidant activity assay based on FIA

Antioxidant activity has been demonstrated to be an effective means of evaluating the biological activity of GE and related products *in vitro*^45,46^. To determine the total antioxidant capacity of GE samples, DPPH assays were performed by using the method introduced by Mrazek *et al*.^36^ with slightly modification. In this study, the total antioxidant activities of the GE samples were assessed by the on-line UV spectroscopic DPPH method, where ascorbic acid equivalent (ASAE) values were measured as shown in Table 1. The results indicated that a majority of the GE samples were found to possess excellent antioxidant activities with an ASAE value above 0.2000 mM; however, S6, S9, S10 and S19 showed lower ASAE values (<0.2000 mM), indicating poor antioxidant activities. In addition, the ASAE values among different GE samples ranged from 0.1365 mM (S6) to 0.2809 mM (S12), showing a 2.06-fold difference in antioxidant activity. By observing chemical fingerprints and evaluation results, the difference in antioxidant activity was not caused by a simple change in the content of one certain component. Therefore, it is necessary to establish a model to investigate the relationship between antioxidant activity and chemical fingerprint.

#### Relationship between HPLC fingerprints and antioxidant activities

**Figure 5:**
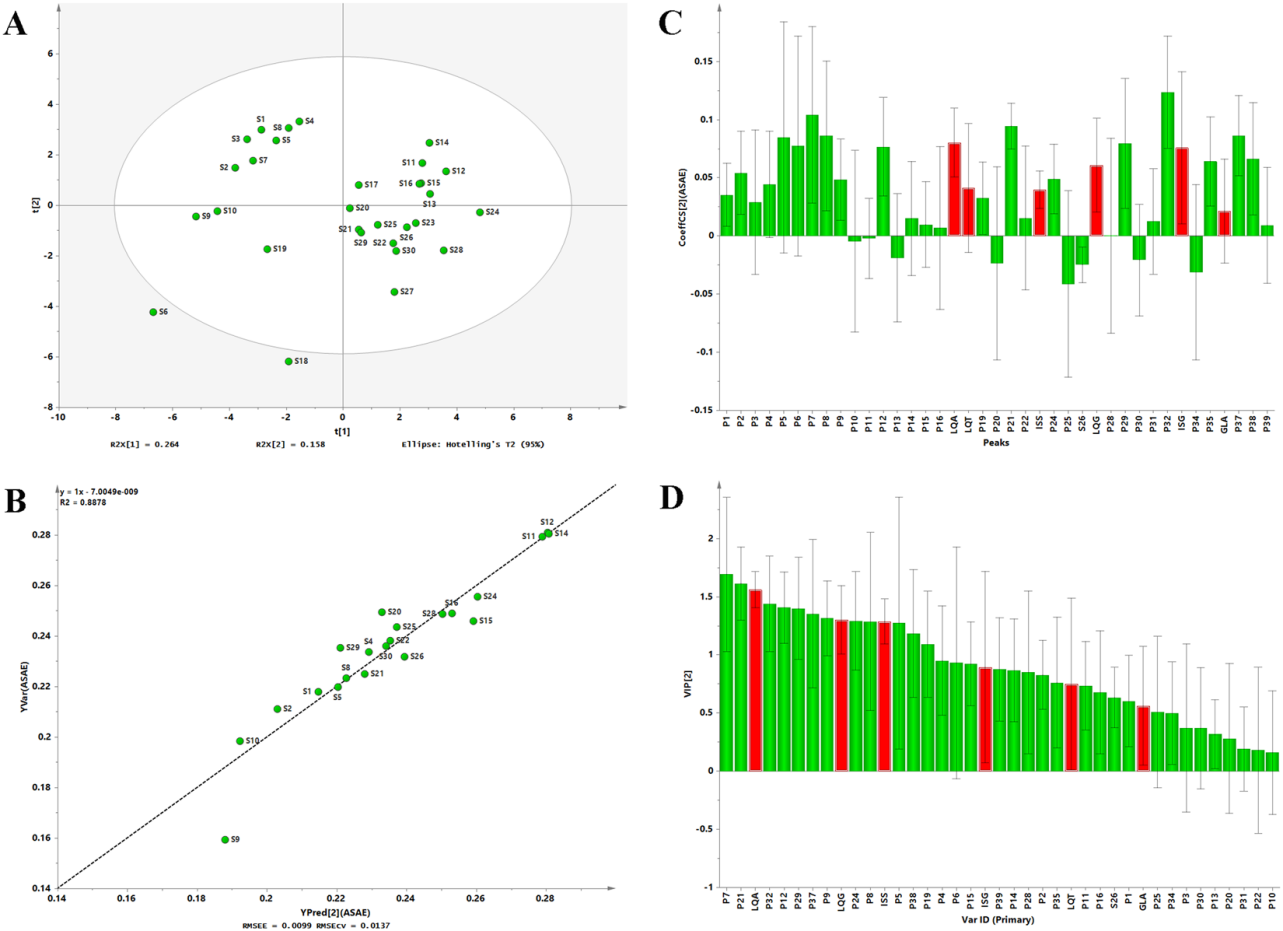
Scores scatter plot (**A**); Y observed versus Y predicted plot for the training set (**B**); standardized regression coefficient plot based on Hotelling’s T2 (0.05) (**C**) and VIP plot (**D**) for GE samples.

A correlation model between the antioxidant activities and HPLC fingerprints was established to discover potential constituents with antioxidant capacity. PLS^47–49^, carried out using SIMCA-P 13.0 software (Umetrics, Sweden), was constructed by the ASAE values as the response matrix *Y*, and the HPLC fingerprints at 220 nm as the descriptor matrix *X* to investigate the spectrum-effect relationship. Based on the score plot centered on the data mean (Fig. 5A), outliers (S6 and S18) were identified and eliminated when constructing the final mathematical model. After excluding the outliers, the rest 28 samples were divided randomly into training and test sets (Table 1) by bootstrap Latin partition method^50,51^. Bootstrap Latin partition method divided 28 GE samples randomly into four equal parts (7 samples in each part), where one randomly part (7 samples) as test set and the other three parts (21 samples) as training set. The established model achieved an explained variance (R^2^) of 88.8%, a predictive ability (Q^2^) of 76.8%, a root mean square error of estimation (RMSEE) and a cross validation procedure (RMSECV) value of 0.0099 and 0.0137, respectively, implying that excellent PLS model was obtained. Furthermore, a satisfactory result with a root mean square error of prediction (*RMSEP*) value of 0.0294 was obtained, suggesting that this model had a robust performance. Regression curve (y = x − 7.0049e^−0.009^, R^2^ = 0.8878, Fig. 5B) was also established by plotting the predicted ASAE values (*x*) *vs*. measured ASAE values (*y*) of 28 batches of GE samples. It indicated that the measured value was consistent with the predicted ASAE one, and the PLS model had a good predictive ability. The standardized regression coefficients plot (Fig. 5C) revealed that the majority of HPLC fingerprint components (including six identified peaks LQA, LQT, ISS, LQG, ISG and GLA) appeared to have positive correlation with the ASAE values, indicating the antioxidants in GE samples might be flavonoids and saponins. The overall contribution of each variable to the established model was reflected by variable influence on projection statistics (VIP) values, the larger VIP values (VIP value > 0.5 usually used as a threshold value), the more relevant for variable classification. Therefore, the six investigated compounds showed a strong antioxidant activity. In particular, the three largest variables (VIP value > 1.5) responsible for positive coefficient with antioxidant activity were the change in peak 7 (VIP value = 1.6928), peak 21 (VIP value = 1.6148) and LQA (VIP value = 1.5607) concentration (Fig. 5D).

## Conclusion

To evaluate the quality consistency of GE samples, this study established a multi-prong approach including quantitative fingerprint evaluation (ALQFM), chemometric methods (HCA) and antioxidant activity assay. In quantitative fingerprint evaluation, all the GE samples showed similar ${S}_{L}$ and $\alpha$; however, ${P}_{L}$ were able to identify the differences among the different samples due to the variations in the chemical contents. Moreover, ${P}_{L}$ was highly correlated with the content of the six investigated compounds, indicating that ALQFM had the potential to replace quantitative analysis to quantify complex HM systems. HCA had the ability to distinguish GE samples from different sources; however, the active ingredients in GE samples could not be clearly indicated. Therefore, with the help of chemometric approaches (PLS), fingerprint-efficacy relationship obtained by the rapid spectroscopic DPPH method was established for exploring the possible anti-oxidant active components in GE sample. The strategy proposed in this study provides a powerful, effective and practical method to evaluate the quality consistency of GE samples.

## Supplementary information

Supplementary Information
