# Supplementary material for: A strategy for qualitative and quantitative profiling of glycyrrhiza extract and discovery of potential markers by fingerprint-activity relationship modeling
Source: Sci Rep. 2019 Feb 4;9:1309. doi: 10.1038/s41598-019-38601-y (PMC6361909; doi:10.1038/s41598-019-38601-y)
Supplement: Supplementary file 1 — Supplementary Information [file 41598_2019_38601_MOESM1_ESM.docx]

**Supplementary Information**

**A strategy for qualitative and quantitative profiling of glycyrrhiza extract and discovery of potential markers by fingerprint -activity relationship modeling**

Yujing Zhang^1^, Chao Wang^2^, Fangliang Yang^1^, Guoxiang Sun^1*^

^1^ School of Pharmacy, Shenyang Pharmaceutical University, Shenyang, Liaoning, P. R. China.

^2^ School of Pharmaceutical Engineering, Shenyang Pharmaceutical University, Shenyang, Liaoning, P. R. China.

**Contents**

[1 Supplementary Figures 3](#_Toc532239770)

[**Figure S1** HPLC fingerprints of 30 batches of GE samples at five wavelengths. 3](#_Toc532239771)

[**Figure S2.** Correlation between ***P*_L_** and ***P*_6C_** of 30 batches of GE samples. 4](#_Toc532239772)

[**Figure S3** Histogram of quality grade difference between the fusion and single wavelength fingerprint. 5](#_Toc532239773)

[2 Supplementary Tables 6](#_Toc532239774)

[**Table S1** TCM/HM quality grades criteria based on ALQFM. Samples with the grade≤5 are recommended as the qualified ones. 6](#_Toc532239775)

[**Table S2** Calibration curves, LOD and LOQ of six investigated compounds. LQA, liquiritin apioside; LQT, liquiritin; LQG, liquiritigenin; GLA, glycyrrhizic acid; ISS, isoliquiritoside; ISG, isoliquiritigenin. 7](#_Toc532239776)

1 Supplementary Figures

| 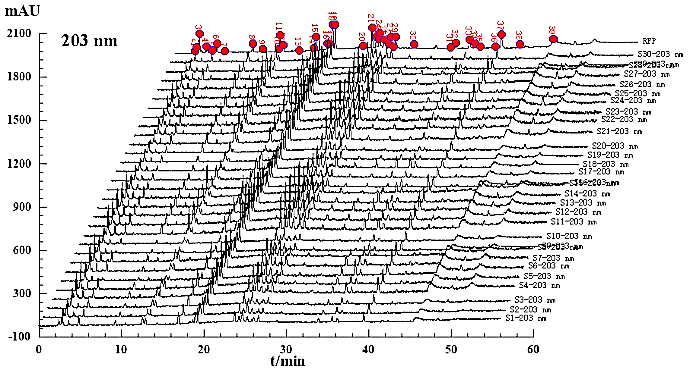 | 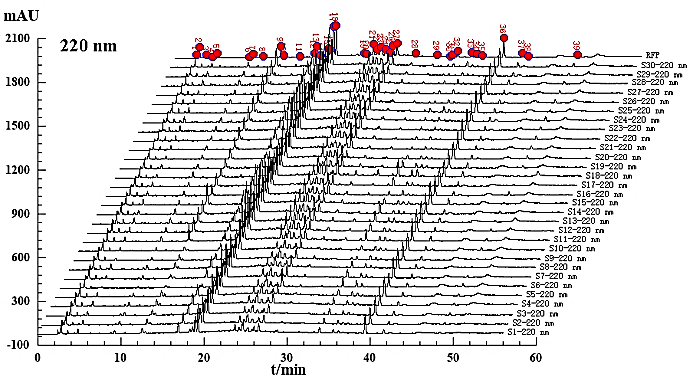 |
| --- | --- |
| 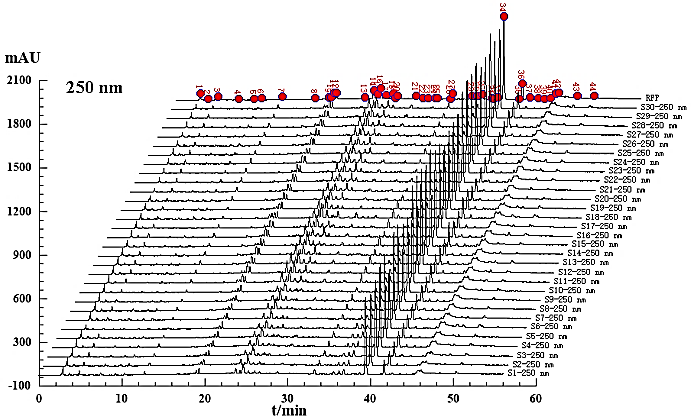 | |
| 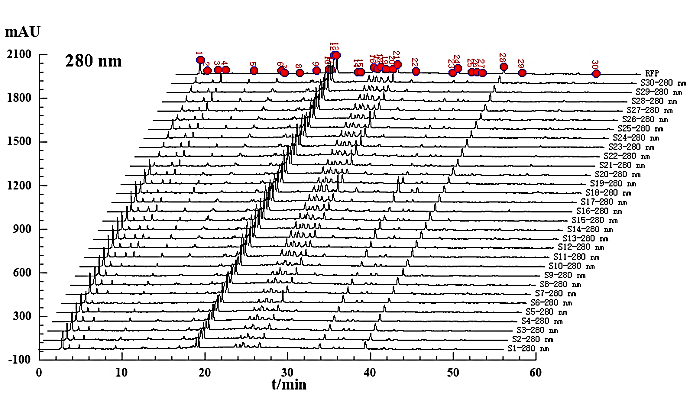 | 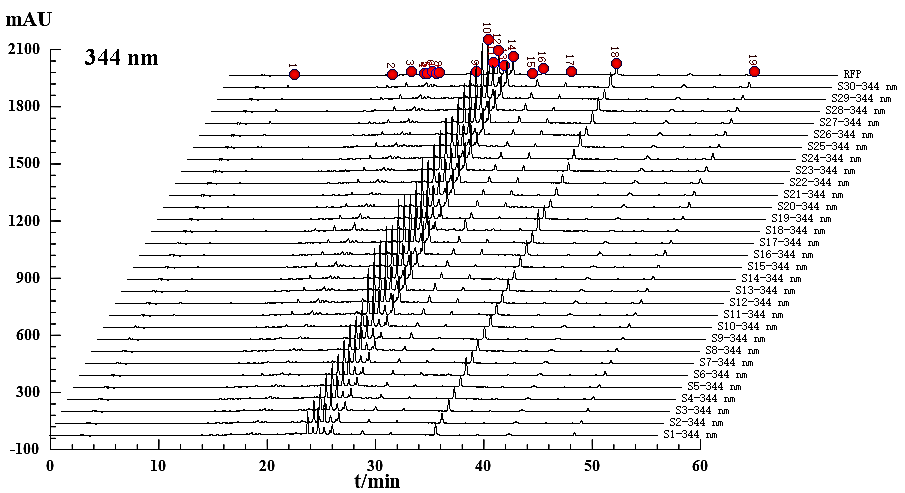 |

**Figure S1** HPLC fingerprints of 30 batches of GE samples at five wavelengths.

**
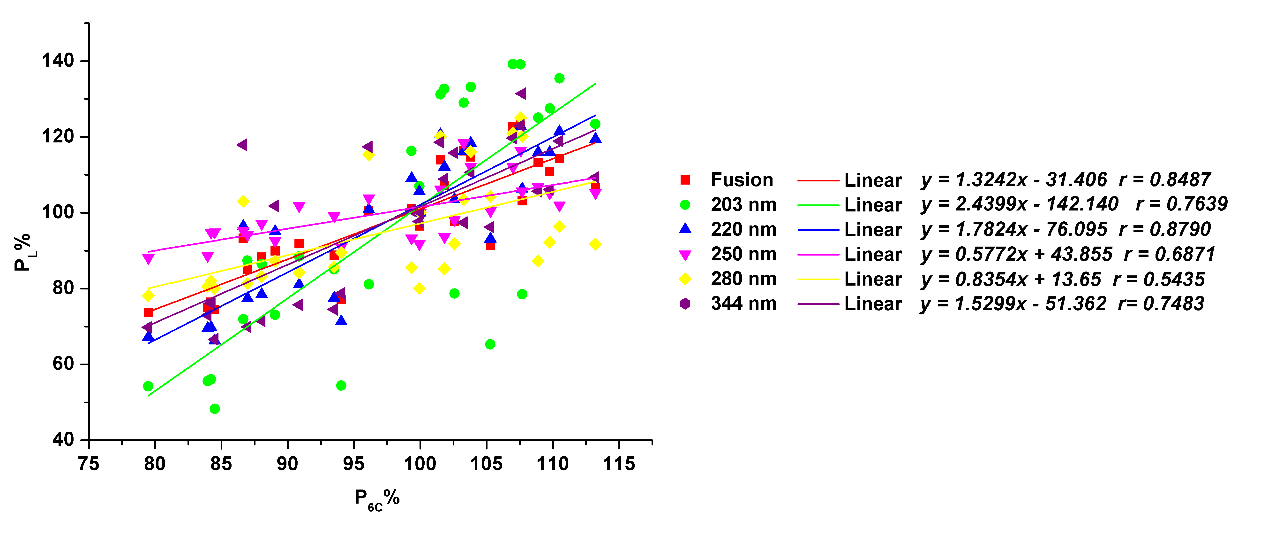
**

**Figure S2.** Correlation between ***P*_L_** and ***P*_6C_** of 30 batches of GE samples.

**Figure S3** Histogram of quality grade difference between the fusion and single wavelength fingerprint.

2 Supplementary Tables

**Table S1** TCM/HM quality grades criteria based on ALQFM. Samples with the grade≤5 are recommended as the qualified ones.

| Grade | 1 | 2 | 3 | 4 | 5 | 6 | 7 | 8 |
| --- | --- | --- | --- | --- | --- | --- | --- | --- |
| *S*_L_≥ | 0.95 | 0.9 | 0.85 | 0.8 | 0.7 | 0.6 | 0.5 | ***S*_L_**＜0.5 |
| *P*_L_∈ | 95~105 | 90~110 | 80~120 | 75~125 | 70~130 | 60~140 | 50~150 | 0~∞ |
| *α*≤ | 0.05 | 0.10 | 0.15 | 0.20 | 0.30 | 0.40 | 0.50 | α﹥0.05 |
| Quality | best | better | good | fine | moderate | common | inferiors | defective |

**Table S2** Calibration curves, LOD and LOQ of six investigated compounds. LQA, liquiritin apioside; LQT, liquiritin; LQG, liquiritigenin; GLA, glycyrrhizic acid; ISS, isoliquiritoside; ISG, isoliquiritigenin.

| Compound^a)^ | Regression equation^b)^ | R^2^ | Linear range (μg/mL) | LOD^c)^ (μg/mL) | LOQ^d)^ (μg/mL) |
| --- | --- | --- | --- | --- | --- |
| LQA (220 nm) | *y* = 13.450*x* – 15.02 | 0.9999 | 10.00-400.0 | 0.100 | 0.500 |
| LQT (220 nm) | *y* = 15.726*x* + 62.18 | 0.9999 | 5.000-500.0 | 0.125 | 5.000 |
| ISS (344 nm) | *y* = 29.985*x* + 1.65 | 1.0000 | 0.500-50.00 | 0.025 | 0.500 |
| LQG (220 nm) | *y* = 65.135*x* + 194.37 | 0.9999 | 1.000-200.0 | 0.050 | 1.000 |
| ISG (344 nm) | *y* = 28.312*x* + 9.69 | 1.0000 | 2.000-200.0 | 0.100 | 2.000 |
| GLA (250 nm) | *y* = 7.140*x* + 42.37 | 0.9999 | 120.0-1200 | 0.600 | 3.000 |

1. LQA, Liquiritin apioside; LQT, Liquiritin; ISS, Isoliquiritoside; LQG, Liquiritigenin; ISG, Isoliquiritigenin; GLA, Glycyrrhizic acid.
2. *y* is the peak area, *x* is the concentration injected (μg/mL).
3. LOD: limit of detection (S/N=3).
4. LOQ: limit of quantification (S/N=10).
